# Small-Molecule Organocatalysis Facilitates In Situ Nucleotide Activation and RNA Copying

**DOI:** 10.1021/jacs.3c04635

**Published:** 2023-07-11

**Authors:** Harry R. M. Aitken, Tom H. Wright, Aleksandar Radakovic, Jack W. Szostak

**Affiliations:** † Howard Hughes Medical Institute, Massachusetts General Hospital, Boston, Massachusetts 02114, United States; ‡ Department of Molecular Biology, Massachusetts General Hospital, Boston, Massachusetts 02114, United States; § Center for Computational and Integrative Biology, Massachusetts General Hospital, Boston, Massachusetts 02114, United States; ∥ Department of Genetics, Harvard Medical School, Boston, Massachusetts 02115, United States; ⊥ Department of Chemistry and Chemical Biology, Harvard University, Cambridge, Massachusetts 02138, United States; # Department of Chemistry, Howard Hughes Medical Institute, University of Chicago, Chicago, Illinois 60637, United States

## Abstract

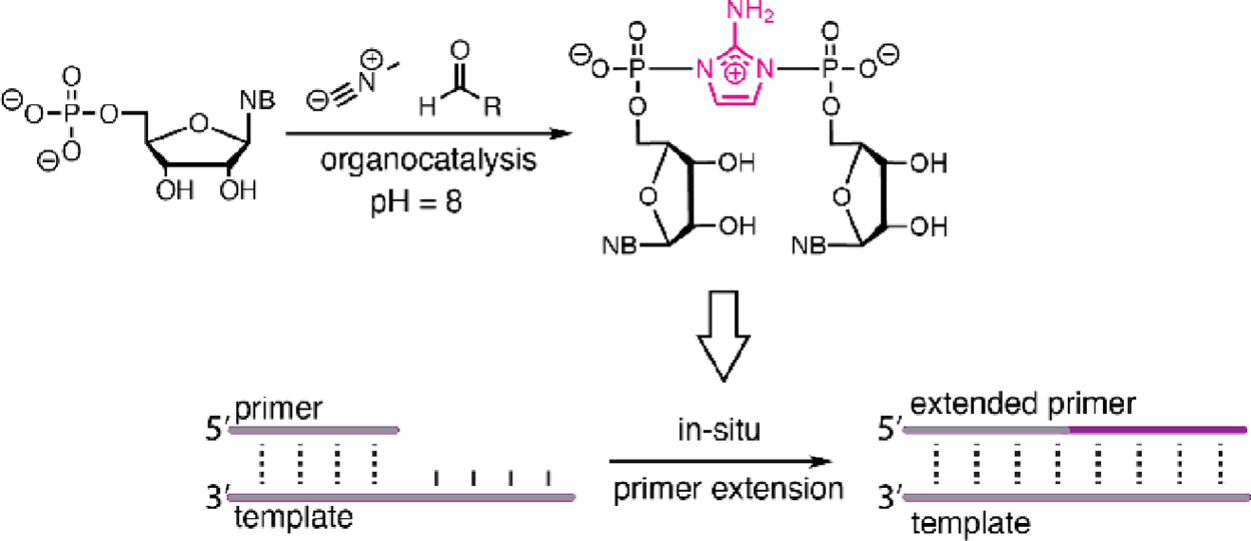

A key challenge in origin-of-life research is the identification of plausible conditions that facilitate multiple steps along the pathway from chemistry to biology. The incompatibility of nucleotide activation chemistry and nonenzymatic template-directed RNA copying has hindered attempts to define such a pathway. Here, we show that adding heteroaromatic small molecules to the reaction network facilitates in situ nucleotide phosphate activation under conditions compatible with RNA copying, allowing both reactions to take place in the same mixture. This is achieved using Passerini-type phosphate activation in concert with nucleophilic organocatalysts that intercept high-energy reactive intermediates; this sequence ultimately affords 5′,5′-imidazolium-bridged dinucleotides—the active species in template-directed RNA polymerization. Our results suggest that mixtures of prebiotically relevant heteroaromatic small molecules could have played a key role in the transition from chemistry to biology.

## Introduction

**Figure 1:**
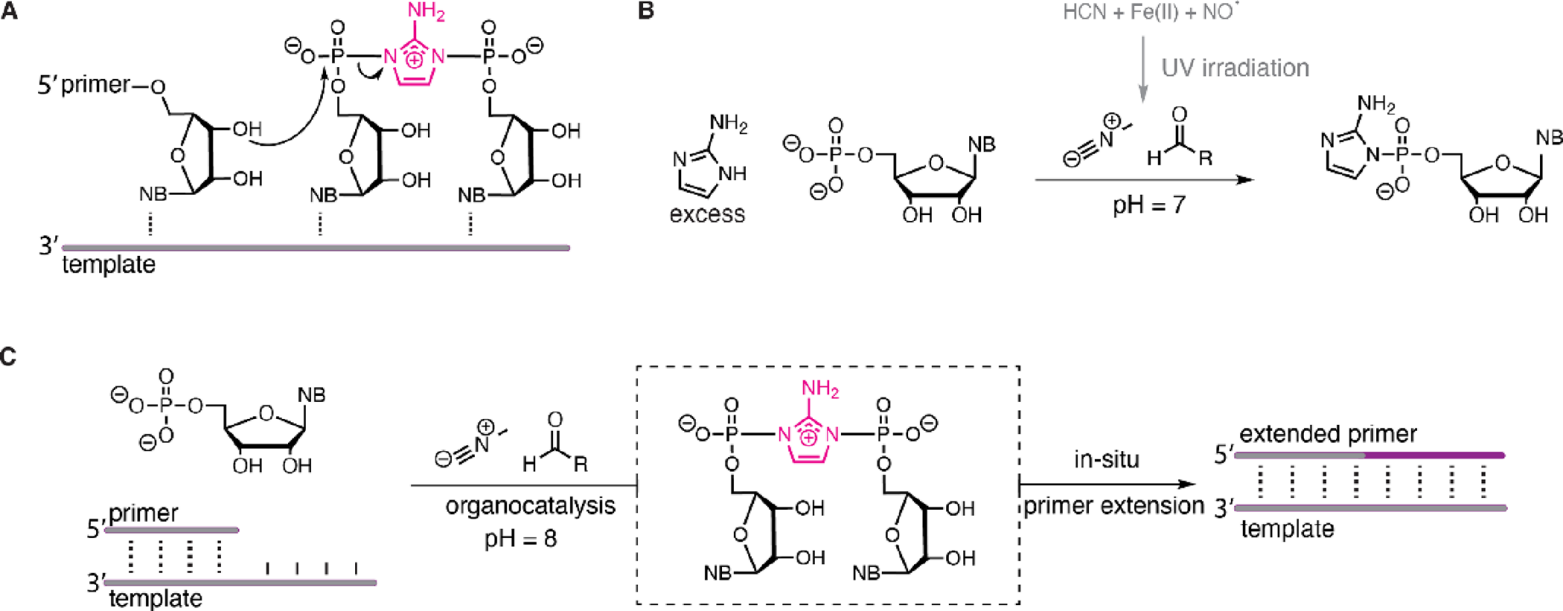
(A) Primer extension via an imidazolium-bridged dinucleotide. (B) Sutherland et al.’s Passerini-type nucleotide activation.^10^ (C) This work, combining organocatalyzed activation with in situ RNA copying. (B) is adapted from Mariani, A.; Russell, D. A.; Javelle, T.; Sutherland, J. D. A Light-Releasable Potentially Prebiotic Nucleotide Activating Agent. *J. Am. Chem. Soc.***2018**, 140 (28), 8657–8661. Copyright 2018 American Chemical Society.

The RNA world is a prominent hypothesis for the advent of life on Earth. This model posits an early stage of life in which RNA sequences acted as both genetic polymers and, when folded appropriately, as ribozyme catalysts that enabled simple metabolic pathways.^1,2^ Prior to the emergence of the first ribozymes, however, nucleotide activation and RNA replication must have depended on primitive chemical processes.^3^ Experiments that mimic primordial nonenzymatic RNA oligomerization typically employ spontaneous templated copying of nucleoside 5′-phosphorimidazolides, a process first pioneered by Leslie Orgel in 1968.^4−6^ More recently, our laboratory has discovered enhanced copying of RNA templates using 2-aminoimidazole (2AI) activated nucleotides (2AI-pN, or *N),^7^ which equilibrate in solution to form 5′,5′-imidazolium-bridged dinucleotides (N*N), the active species in template-directed polymerization (Figure 1A).^8,9^

In 2018, the Sutherland lab reported the first prebiotically plausible synthesis of 5′-phosphorimidazolides, by activating nucleoside monophosphates (NMPs) with methyl isocyanide and acetaldehyde following a Passerini-type mechanism (Figure 1B).^10^ This chemistry is initiated by simple aldehydes and methyl isocyanide, which may be derived from methylamine by ultraviolet irradiation in a ferrocyanide- and nitroprusside-rich environment. Although this work addresses a major challenge in mapping a pathway from prebiotic chemistry to the RNA world, the conditions employed were not directly compatible with RNA copying. When activation was performed with 2AI, it required a large excess of 2AI to intercept the hydrolytically labile imidoyl phosphate intermediate. However, excess 2AI drives the equilibrium between 2AI-pN and imidazolium-bridged dinucleotide (N*N) toward the mononucleotide, preventing the accumulation of the active species and suppressing copying.^11^ More recently, our laboratory found that freeze–thaw cycles—in which reactants are concentrated in a eutectic phase—facilitate methyl isocyanide-initiated nucleotide activation with low 2AI concentrations.^12,13^ While this approach builds on Sutherland et al.’s work to enable one-pot combined in situ activation and RNA copying, it introduces new complications. Most pertinently, freeze–thaw cycles disrupt vesicle membranes and lead to the exchange of contents between vesicles.^14,15^ It is unclear whether protocells could form and evolve under these conditions. In addition, the use of freeze–thaw cycles limits the compatible geochemical scenarios.

Here, we report a new catalytic pathway that combines prebiotically plausible nucleotide activation with in situ RNA copying, enabling simultaneous isocyanide-mediated activation and templated oligonucleotide polymerization in solution. Specifically, we report that a variety of heteroaromatic small molecules act as organocatalysts by intercepting high-energy intermediates and forming semi-stabilized adducts with moderated reactivity. This diverted reaction sequence suppresses hydrolysis and facilitates substitution with relatively low concentrations of 2AI, ultimately generating 5′,5′-imidazolium-bridged dinucleotides under standard RNA copying conditions (Figure 1C).

The small molecules used in this chemistry are plausible components of the primordial milieu, many of which are readily formed from simple prebiotic precursor feedstocks through cyanosulfidic photoredox chemistry^16−18^ or even in Miller–Urey spark discharge experiments.^19−21^ Notably, this use of nucleophilic organocatalysis is conceptually similar to a reaction network reported by Richert’s group, in which 1-ethylimidazole modulates carbodiimide-mediated phosphate activation and facilitates the synthesis of chimeric amino acid–nucleic acid conjugates, among other primitive biomolecules.^22^

## Results and Discussion

**Scheme 1:**
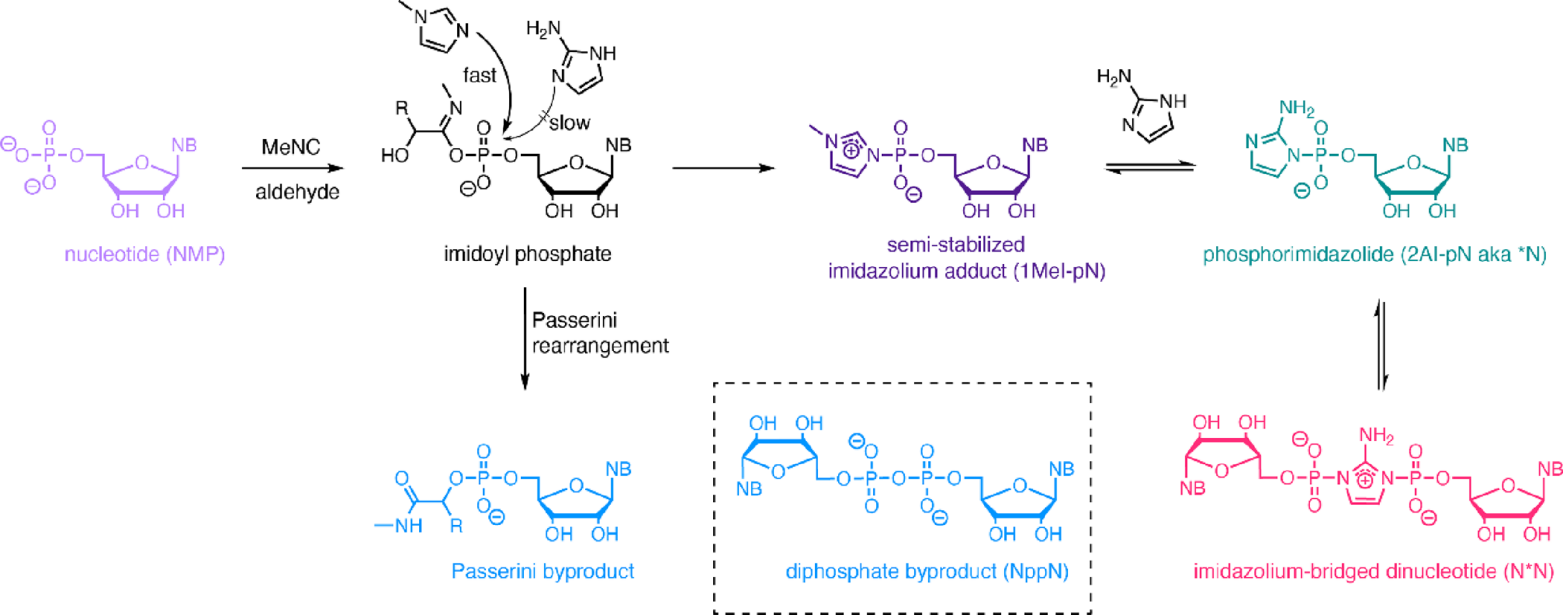
1-Methylimidazole-Catalyzed Synthesis of Imidazolium-Bridged Dinucleotide (N*N)

Passerini-type isocyanide-mediated nucleotide activation chemistry likely proceeds via an imidoyl phosphate intermediate. This highly reactive species reacts efficiently with stoichiometric imidazole, but a large excess of 2AI is necessary to compete with hydrolysis and provide a high yield of 2AI-activated nucleotide.^10,23^ Given that 2AI (p*K*_a_ = 8.5) is a stronger nucleophile than imidazole (p*K*_a_ = 7.0), we reasoned that the primary barrier to a high-yielding reaction with 2AI was its relative availability due to its competing protonation in solution. Moreover, as the reaction proceeds cleanly with imidazole, we wondered whether it might also be possible for weaker nucleophiles to intercept the reactive imidoyl phosphate moiety, forming a semi-stabilized adduct activated at the 5′-phosphate that would be less prone to hydrolysis and byproduct formation (Scheme 1). We postulated that for some nucleophiles, this species might still be readily substituted with 2AI and therefore participate in an equilibrium with the 5′-phosphorimidazolide-activated monomer (2AI-pN or *N) and the 5′,5′-imidazolium-bridged dinucleotide N*N. A range of small molecules could potentially catalyze this reaction pathway, but we initially focused on the use of 1-methylimidazole (1MeI) (p*K*_a_ = 7.4) because it has a similar nucleophilicity to imidazole but is a better leaving group at high pH due to the permanent positive charge on the imidazolium ring. Moreover, 1MeI has previously been employed as a catalyst in prebiotic RNA ligation experiments^24^ and is a plausible constituent of the methylamine-rich environment^25^ required to generate methyl isocyanide.

**Figure 2:**
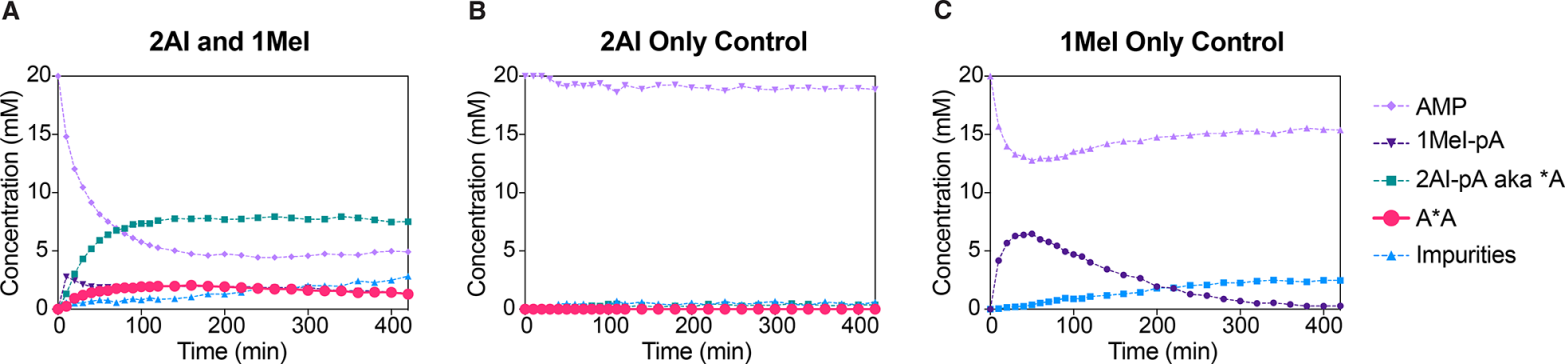
^31^P NMR time course charts for Passerini-type adenosine monophosphate activation. Standard conditions: AMP (20 mM), MgCl_2_ (30 mM), HEPES (200 mM, pH = 8), initiated with MBA/MeNC (200 mM each). (A) With 1MeI (50 mM); (B) with 2AI (10 mM); (C) with 1MeI (50 mM) and 2AI (10 mM).

We began by subjecting 20 mM adenosine monophosphate (AMP) to activation with methyl isocyanide and 2-methylbutyraldehyde at room temperature in the presence of 50 mM of 1MeI and 10 mM of 2AI under typical RNA copying conditions (pH = 8; 30 mM of MgCl_2_). 2-Methylbutyraldehyde was chosen in these initial experiments, as it has been shown to suppress Passerini byproduct formation in isocyanide-mediated activation, relative to simpler aldehydes.^23^ Under these conditions, we were gratified to observe the rapid appearance of the imidazolium adduct 1MeI-pA, followed by slow formation of both the 2AI-activated monomer *A and the imidazolium-bridged dinucleotide A*A (Figure 2A). The dinucleotide A*A formed in concentrations up to 2.0 mM after 2 h. In comparison, a control experiment without 1MeI resulted in minimal consumption of AMP, and the reaction afforded only trace levels of the activated monomer *A and the 5′,5′-diphosphate byproduct AppA (Figure 2B). Conversely, when activation was performed without 2AI, imidazolium adduct 1MeI-pA rapidly accumulated in solution up to 6.5 mM but was then slowly hydrolyzed to AMP and also converted to the 5′,5′-diphosphate byproduct AppA (Figure 2C). Taken together, these results support the proposed mechanism: first, 1MeI rapidly intercepts a reactive imidoyl phosphate intermediate to form an imidazolium-activated monomer, 1MeI-pA. As 1MeI is a better leaving group than 2AI, this species is slowly substituted, leading to an equilibrium population of 1MeI-pA, phosphorimidazolide 2AI-pA, and bridged dinucleotide A*A. Among the undesired side products, AppA was generated from 5′-phosphate attack on activated nucleotide species and its rate of formation was correlated with the concentration of 1MeI in solution, suggesting that substitution of 1MeI-pA was particularly efficient. The Passerini byproduct was only observed in the control experiment without 1MeI. Addition of 1MeI effectively suppressed formation of this species, suggesting that rapid nucleophilic attack of 1MeI outcompetes the intramolecular Passerini rearrangement.

**Figure 3:**
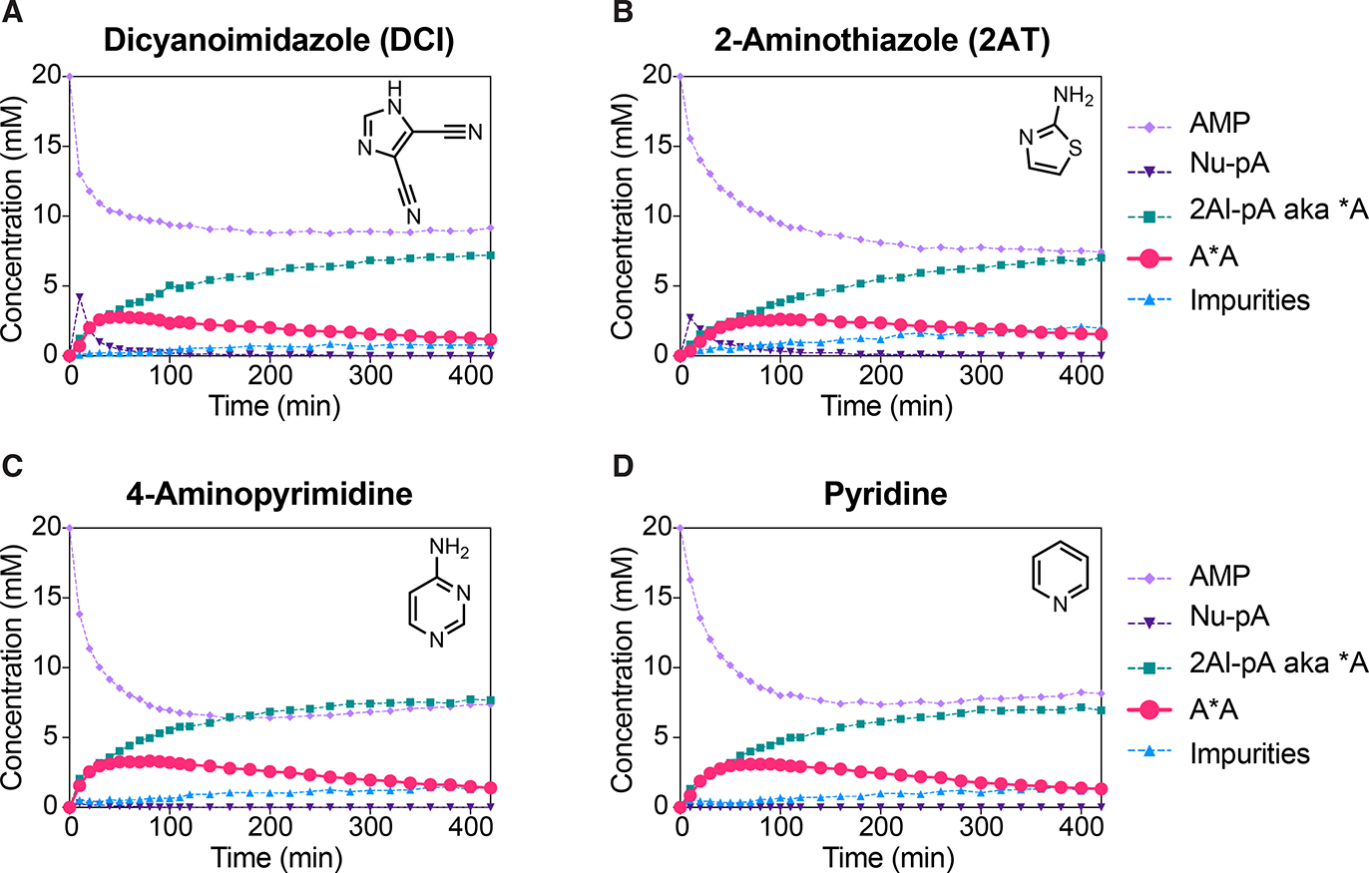
^31^P NMR time course charts for Passerini-type adenosine monophosphate activation. Standard conditions: AMP (20 mM), 2AI (10 mM), organocatalyst (50 mM), MgCl_2_ (30 mM), HEPES (200 mM, pH = 8), initiated with MBA/MeNC (200 mM each). Nu-pA = adduct of nucleophilic organocatalyst and 5′-phosphate activated AMP.

Next, we asked whether we could generalize the organocatalysis of isocyanide-mediated phosphate activation to other nucleophilic small molecules. For instance, 4,5-dicyanoimidazole (DCI) has been implicated in several roles in prebiotic chemistry, including as a catalyst in phosphate and amino acid activation^26^ and as an intermediate in purine nucleoside synthesis.^17,27^ On the other hand, 2-aminothiazole (2AT) has been shown to play roles in primordial nucleoside synthesis and may have shared a common origin with 2AI.^17,28^ We chose to study these compounds, together with other simple heterocycles including 4-aminopyrimidine and pyridine (Figure 3). Gratifyingly, all of these nucleophiles efficiently catalyzed the formation of imidazolium-bridged dinucleotide A*A at concentrations of 2.7–3.3 mM (peak concentration) under Passerini-type activation conditions. Moreover, as these weak nucleophiles are all better leaving groups than 1MeI, the phosphate adducts of these species did not persist in solution. For example, when activation was catalyzed by DCI, the concentration of the intermediate adduct DCI-pA was 4.0 mM within the first minutes of the reaction but rapidly dropped to <0.1 mM after 2 h.

**Figure 4:**
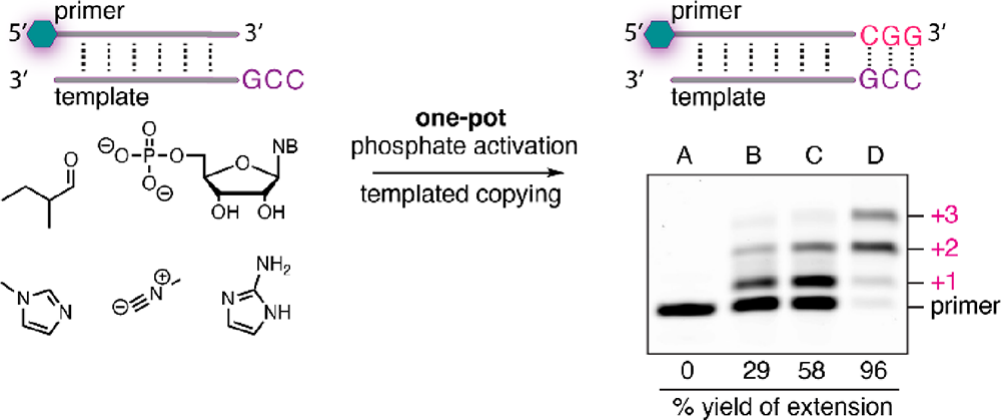
Schematic of Passerini-type in situ nucleotide activation and RNA copying results for in situ organocalyzed imidazolium-bridged dimer formation and control experiments. A: In situ activation with only 2AI; B: in situ activation and copying with only 1MeI; C: in situ activation and copying with both 1MeI and 2AI; D: copying with presynthesized, purified *C and *G.

Encouraged by these results, we sought to combine this mode of organocatalyzed nucleotide activation with in situ RNA copying. To do so, we first tested template copying using a BODIPY dye-labeled primer base-paired to a model template sequence with an unpaired 5′-CCG-3′ overhang (Figure 4). Using the same activation conditions reported in Figure 2C (i.e., in the presence of 1MeI and 2AI) with CMP and GMP (10 mM each), the copying proceeded in up to 58% yield (+1 or higher primer extension) after 24 h. When in situ activation and copying were conducted *without* 2AI in the reaction mixture, primer extension was still observed in 29% yield (+1 or higher) after 24 h. In this experiment, copying likely proceeds *via* 3′-OH attack on a 1MeI-pN activated nucleotide. Similar copying with monomeric substrates has been associated with an error frequency higher than those of extension reactions with imidazolium-bridged dinucleotides.^29^ No primer extension was observed when 1MeI was excluded from the reaction mixture, while a positive control using presynthesized and purified 2AI-activated monomers *C and *G resulted in the most efficient copying, as expected.

To further examine the substrate scope of this copying, we next turned to activation and copying with alternative nucleophilic organocatalysts including DCI (**2**), 2AO (**3**), 2AT (**4**), pyrimidines (**5**–**7**), and pyridines (**8**–**13**). In the presence of 2AI, these species all catalyzed in situ imidazolium-bridged dimer N*N formation and concurrent primer extension (Table 1). These results revealed a trend that activation works best when the nucleophile has a p*K*_a_ of ∼5–6. Indeed, among the species tested 2AT (p*K*_a_ = 5.3), DCI (p*K*_a_ = 5.2), and 4-aminopyrimidine (p*K*_a_ = 5.7) facilitated the highest yield of primer extension. This trend was also borne out when comparing copying with a range of 4-substituted pyridines. The sole exception was with 4-fluoropyridine, which caused precipitation of the reaction mixture without affording any primer extension product. Taken together, these results suggest that nucleophilicity of the small molecule must be balanced to optimize N*N formation and copying: the species must be sufficiently reactive to intercept the imidoyl phosphate and outcompete hydrolysis, but not so reactive that it decomposes the imidazolium-bridged dimer. In addition, no copying was observed in the absence of 2AI, suggesting that the nucleotide adducts of the nucleophilic organocatalysts (Nu-pN) do not directly participate in copying. This is particularly intriguing given that Ferris and co-workers have shown that dimethylaminopyridinium (DMAP)-activated adenosine 5′-phosphate—the same type of activated nucleotide that we observe as an intermediate in activation catalyzed by DMAP **13**—can undergo surface-catalyzed oligomerization on montmorillonite.^30^

**Table 1:**
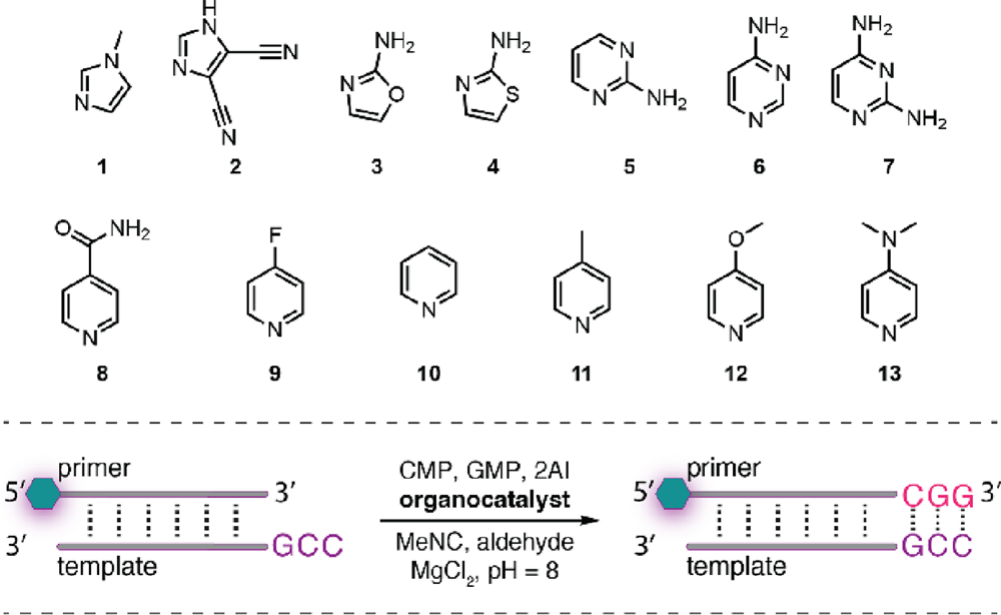
Results of In Situ Activation and RNA Copying Catalyzed by Heteroaromatic Small Molecules

number	organocatalyst^a^	p*K*_a_^b^	yield (%, ≥+1)^c^
**1**	1-methylimidazole	7.4	*58*^d^
**2**	4,5-dicyanoimidazole	5.2^e^	68
**3**	2-aminooxazole	4.6	50
**4**	2-aminothiazole	5.3	65
**5**	2-aminopyrimidine	3.5	16
**6**	4-aminopyrimidine	5.7	57
**7**	2,4-diaminopyrimidine	7.3	55
**8**	isonicotinamide	3.6	34
**9**	4-fluoropyridine	4.6	0^f^
**10**	pyridine	5.2	42
**11**	4-methylpyridine	6.0	35
**12**	4-methoxypyridine	6.6	33
**13**	4-dimethylaminopyridine	9.7	19

a Standard conditions: CMP (10 mM), GMP (10 mM), 2AI (10 mM), organocatalyst (50 mM), MgCl_2_ (30 mM), HEPES (200 mM, pH = 8), initiated with MBA/MeNC (200 mM each).

b p*K*_a_ of the conjugate acid of the catalyst, unless noted.

c Yield reported for the proportion of primer that extends by one residue (or more) in the presence of 2AI, after 24 h. No copying was observed without 2AI in solution, unless noted.

d Activation and copying with 1MeI only (i.e., without 2AI) afforded 41% primer extension.

e p*K*_a_ of 4,5-dicyanoimidazole.

f Reaction mixture precipitates.

Given our still limited understanding of the geochemical constraints on the primordial Earth, a desirable feature of prebiotic chemistry is robustness to environmental fluctuations.^17,31^ Therefore, we sought to parametrize the activation/copying conditions, by varying the concentration of nucleotide monophosphate, nucleophilic catalyst, and 2AI. Although the optimal concentration of each reaction component is dependent on the exact properties and structure of the organocatalyst used, we chose to avoid this complication by studying activation robustness in aggregate, employing a mixture of 2AT/DCI/4-aminopyrimidine (1:1:1) as the nucleophilic organocatalyst. One benefit of this approach is that it mimics a more realistic scenario where relatively low concentrations of several related small molecules are present and react in concert. The results of these experiments are summarized in Table 2. Although nucleophilic additives are a prerequisite for in situ activation, some erosion in primer extension yield was observed at higher concentrations, due to the propensity of the nucleophiles to catalyze hydrolysis and decompose the imidazolium-bridged dinucleotide (entry 4). Varying the starting concentration of NMP (10–30 mM) or 2AI (5–15 mM) had a modest effect on overall copying while the copying efficiency was significantly reduced at lower pH (entries 5 to 10). Next, we found that lowering the concentrations of methyl isocyanide and 2-methylbutyraldehyde (to 100 mM each) moderately reduced the yield of primer extension, affording 47% +1 and higher products (entry 11).

**Table 2:**
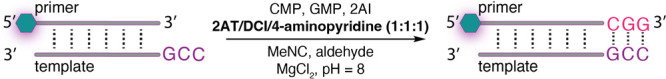
Conditions Screen for In Situ Activation and Copying Catalyzed by 2AT/DCI/4-Aminopyrimidine (1:1:1)

entry	variation from standard^a^	yield (%, ≥+1)^b^
**1**	standard	65
**2**	no nucleophilic catalyst	0
**3**	less catalyst (25 mM)	66
**4**	more catalyst (75 mM)	50
**5**	less 2AI (5 mM)	49
**6**	more 2AI (15 mM)	50
**7**	less nucleotide (5 mM each)	33
**8**	more nucleotide (15 mM each)	56
**9**	pH = 7.5	40
**10**	pH = 7.0	13
**11**	less MeNC/MBA (100 mM)	47
**12**	acetaldehyde instead of MBA	47
**13**	glycolaldehyde instead of MBA	41
**14**	low temperature (−3 °C)	87
**15**	low temperature with acetaldehyde	85

a Standard conditions: CMP (10 mM), GMP (10 mM), 2AI (10 mM), 2AT/DCI/4-aminopyrimidine (1:1:1, 50 mM total), MgCl_2_ (30 mM), HEPES (200 mM, pH = 8), initiated with MBA/MeNC (200 mM each).

b Yield reported for the proportion of primer that extends by one residue (or more) after 24 h.

**Scheme 2:**
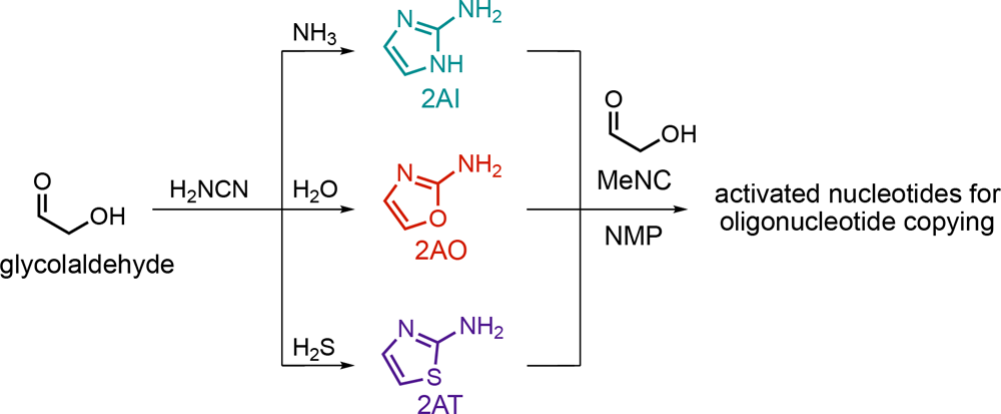
A Mixture of 2AI, 2AO, and 2AT and Their Precursor Glycolaldehyde Work in Tandem with Methyl Isocyanide to Activate Nucleoside Monophosphates

The above experiments, and earlier efforts to combine in situ imidazolium-bridged dinucleotide synthesis and primer extension, all employed methyl isocyanide and 2-methylbutyraldehyde as the initial activating reagents. While a prebiotic synthesis of methyl isocyanide is known, the choice of 2-methylbutyraldehyde was largely driven by chemical optimization, whereby the use of electron-rich aldehydes was shown to reduce the formation of Passerini rearrangement byproducts.^23^ However, when activation is mediated by nucleophilic catalysts, only trace amounts of this Passerini byproduct are observed. Therefore, we next tested activation and copying initiated by more prebiotically relevant aldehydes, such as acetaldehyde^32,33^ and glycolaldehyde,^32−34^ and were gratified to see that they afforded comparable yields of primer extension products (Table 2, entries 12 and 13). Subsequent ^31^P NMR time-course analysis indicated nearly identical levels of imidazolium-bridged dinucleotide A*A formation with acetaldehyde or glycolaldehyde activation, compared to activation with 2-methylbutyraldehyde, albeit with a significant increase in Passerini byproduct formation (0.5 vs 4.5 and 7.0 mM for acetaldehyde and glycolaldehyde, respectively; SI page S9, Figure S3). The successful copying initiated with glycolaldehyde is particularly pleasing as glycolaldehyde is a common intermediate in the prebiotic syntheses of 2AI, 2AT, and 2AO, the latter of which has been implicated as an intermediate in prebiotic nucleoside synthesis.^16,35^ It is striking that a combination of these related 2-aminoazoles and their precursor, glycolaldehyde, may be sufficient to drive nucleotide activation and template copying upon the introduction of a single reagent: methyl isocyanide (Scheme 2). Although beyond the scope of this work, future experiments will be directed at recapitulating this more complex pathway.

**Figure 5:**
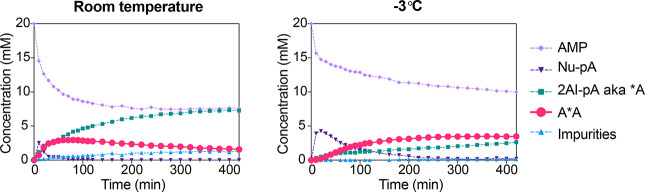
^31^P NMR time-course charts for Passerini-type adenosine monophosphate activation catalyzed by 2AT/DCI/4-aminopyrimidine at room temperature and low temperature (−3 °C). Conditions: AMP (20 mM), 2AI (10 mM), 2AT/DCI/4-aminopyrimidine (1:1:1, 50 mM total), MgCl_2_ (30 mM), HEPES (200 mM, pH = 8), initiated with MBA/MeNC (200 mM each).

When in situ activation was repeated at −3 °C, we observed a remarkable improvement in copying efficiency with either 2-methylbutyraldehyde or acetaldehyde (Table 2, entries 14 and 15). It is important to note that these experiments proceeded in the solution phase, without any freezing or eutectic phase concentration; freezing was suppressed due to the high concentrations of salt and organic solutes. We speculated that the organocatalyzed activation pathway may be more selective as off-pathway reactions are suppressed at low temperatures, which further facilitates the formation and stability of imidazolium-bridged dinucleotides. Consistent with this hypothesis, side-by-side comparison of ^31^P NMR time-course experiments indicated that while overall consumption of AMP was reduced at low temperature, the dimer A*A accumulated in similar overall concentration and was significantly more resistant to hydrolysis, persisting at >3 mM concentration over 10 h (Figure 5). Moreover, formation of the diphosphate AppA and Passerini impurities were suppressed at low temperature.

**Figure 6:**
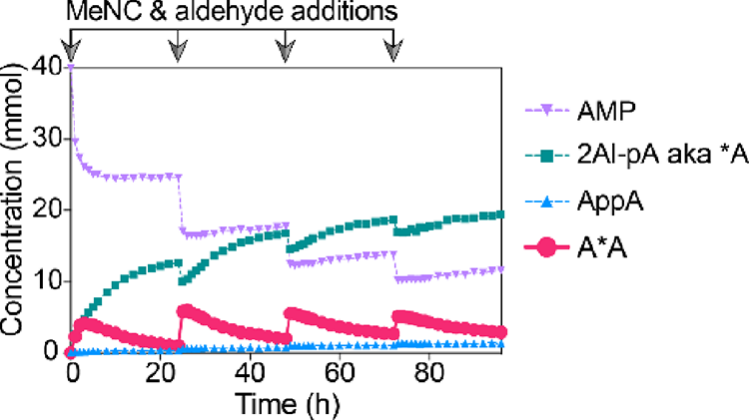
Periodic reactivation of AMP (40 mM) with 2-methylbutyraldehyde/methyl isocyanide (200 mM each, every 24 h) and 2AI (30 mM), catalyzed by 2AT/DCI/4-aminopyrimidine (1:1:1, 50 mM total) at low temperature (−3 °C).

Finally, we sought to test the limits of nucleophilic organocatalyst-modulated in situ activation through multiple rounds of activation and reactivation in order to maintain a high level of activation in the face of reactant hydrolysis. Accordingly, a mixture of AMP (40 mM), 2AI (30 mM), and 2AT/DCI/4-aminopyrimidine (1:1:1) (60 mM total) was subjected to periodic addition of methyl isocyanide/aldehyde every 24 h at −3 °C, resulting in cycles of formation of bridged dinucleotide A*A, together with monomeric *A (Figure 6). Following the addition of each aliquot of methyl isocyanide/aldehyde, AMP was converted to 2AI-pA, while the concentration of bridged dinucleotide A*A fluctuated between 1.2 and 6.0 mM. Throughout the experiment, 5′,5′-diphosphate AppA accumulated as the sole byproduct, reaching a concentration of 1.3 mM after 96 h.

**Figure 7:**
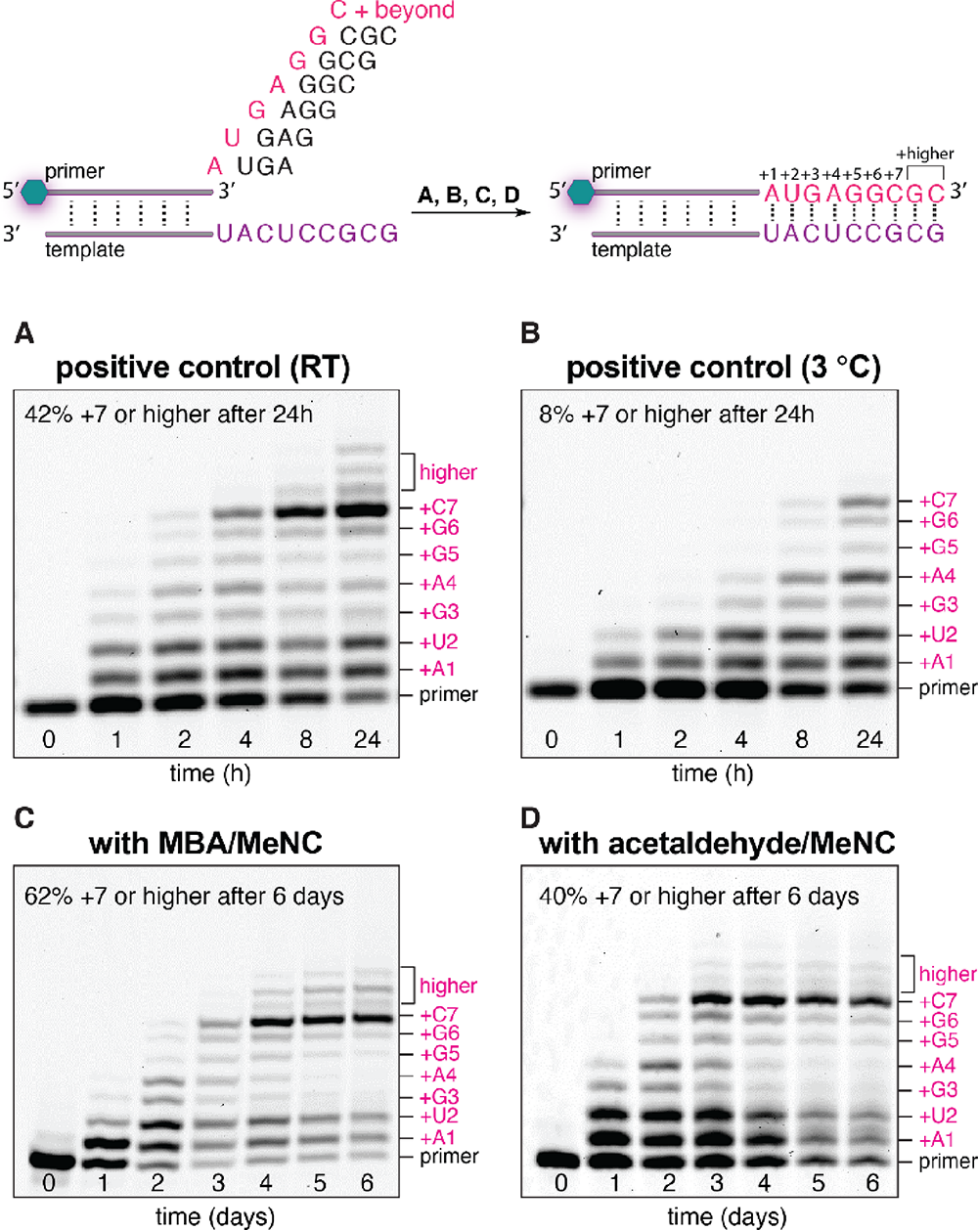
Helper trimer-assisted RNA copying with a template containing all four canonical nucleotides. (A) Positive copying control with preactivated *N monomers and *NNN helper trimers at room temperature. (B) positive copying control at 3 °C. (C) In situ organocatalyzed activation and copying, driven by periodic reactivation of NMPs and trimers with 2-methylbutyraldehyde/methyl isocyanide (200 mM each, every 24 h) at −3 °C. (D) Activation and copying driven by acetaldehyde/methyl isocyanide (200 mM each, every 24 h) at −3 °C.

The cyclical reactivation experiments were then extended to include RNA copying over a complex template sequence incorporating all four canonical nucleotides. Previously, our laboratory has demonstrated that an RNA primer can be extended by seven nucleotides over an unpaired 5′-GCGCCUCAU-3′ template sequence in the presence of six activated helper trimers (pUGA, pGAG, pAGG, pGGC, pGCG, pCGC).^7,12^ Employing this system, two control copying experiments were conducted with preactivated, purified 2AI-activated 5′-phosphorimidazolide monomers and helper trimers (*N and *NNN at 10 mM and 500 μM each, respectively) in the presence of 30 mM MgCl_2_ and annealed primer–template complex, at either room temperature or 3 °C (Figure 7A,B). These experiments revealed that the efficiency of templated copying was reduced at low temperature, suggesting that the stronger template binding of imidazolium-bridged N*NNN species was not sufficient to offset an overall slowdown in RNA copying kinetics, affording only 8% extended primer (+7 nucleotides or higher) after 24 h at 3 °C. Conversely, when copying was combined with in situ organocatalyzed nucleotide activation, optimal results were obtained when the reaction was performed at an even lower temperature. In these experiments, the reaction mixtures were incubated at a colder temperature (i.e., −3 °C) than the positive control, without freezing due to the higher concentration of organic solute. When methyl isocyanide and 2-methylbutyraldehyde (200 mM each every 24 h) were added to a mixture of NMPs (N = A, C, G, U; 10 mM each), unactivated helper trimers (pNNN, 500 μM each), 2AI (30 mM), and a mixture of nucleophilic organocatalysts 2AT/DCI/4-aminopyrimidine (1:1:1) (60 mM total), up to 60% of the primer was extended by seven nucleotides or more (Figure 7C) after 5 days at −3 °C, at which point the whole system appears to stall. The same experiment conducted at room temperature afforded only 7% of fully extended product (Figure S4). Although further experiments are required to determine the precise mechanism of stalling, it is possible that the slowdown in copying was caused by a buildup of denaturing organic solutes and inhibitory byproducts such as AppA. When the organocatalyst-modulated in situ activation was initiated with acetaldehyde—instead of methyl butyraldehyde—copying proceeded more rapidly over the first 72h before stalling at 40% yield (+7 nucleotides or higher; Figure 7D). An exploration of modified conditions showed that copying was quite robust, and similar results were obtained at (i) lower concentrations of methyl isocyanide/2-methylbutyraldehyde (100 mM each every 24 h), (ii) lower concentrations of nucleotides and 2AI (5 mM each and 15 mM, respectively), or (iii) when only 2AT (60 mM) was used as the nucleophilic organocatalyst (Figure S5). Overall, these results show that comparable amounts of fully extended primer can be obtained by template copying under a wide range of conditions. We suggest that a greater extent of template copying might be achieved in a flow system that would allow for the continuous addition of fresh activation reagents together with the removal of inhibitory side products.

## Conclusions

Our results demonstrate that a number of prebiotically relevant heteroaromatic small molecules catalyze Passerini-type activation chemistry of NMPs. Mechanistically, we suggest that these molecules act as weak nucleophiles, intercepting highly labile intermediates to facilitate a pathway that enables the synthesis of imidazolium-bridged dinucleotides and the subsequent template-directed RNA primer extension. Although the present work employed aldehydes and methyl isocyanide as the initial chemical fuel, the organocatalysis reported herein is not necessarily dependent on any particular activation chemistry and may facilitate imidazolium-bridged dinucleotide formation and RNA copying with alternative energy sources that generate high energy but labile phosphate derivatives. Finally, we note that the chemistry we have explored exploits a variety of different but prebiotically relevant compounds, suggesting broad compatibility with competing geochemical scenarios.

## Supporting Information Available

Experimental conditions for ^31^P NMR time course; primer labeling experimental conditions; standard experimental conditions for in situ activation and RNA copying; gel data for in situ activation and RNA copying catalyzed by heteroaromatic small molecules; gel data for conditions screen for in situ activation and copying catalyzed by 2AT/DCI/4-aminopyrimidine (1:1:1); ^31^P NMR time course charts for AMP activation with various aldehydes; gel data for helper trimer-assisted RNA copying at room temperature, driven by periodic reactivation of NMPs and trimers; additional helper trimer assisted RNA copying experiments, driven by periodic reactivation of NMPs and trimers at −3 °C (PDF)
