# Supplementary material for: Small-Molecule Organocatalysis Facilitates In Situ Nucleotide Activation and RNA Copying
Source: J Am Chem Soc. 2023 Jul 11;145(29):16142–9. doi: 10.1021/jacs.3c04635 (PMC10375519; doi:10.1021/jacs.3c04635)
Supplement: Supplementary file 1 — ja3c04635_si_001.pdf [file ja3c04635_si_001.pdf]

Supporting Information

Small Molecule Organocatalysis Facilitates *In Situ* Nucleotide Activation and RNA Copying

Harry R.M. Aitken,[a,b,c,d,e] *Tom H. Wright,*[a,b,c,d] *Aleksandar Radakovic,*[a,b,c,d] *and Jack W. Szostak**[a,b,c,d,e,f]

a Howard Hughes Medical Institute, Massachusetts General Hospital, Boston, MA 02114, United States

b Department of Molecular Biology, Massachusetts General Hospital, Boston, MA 02114, United States

c Center for Computational and Integrative Biology, Massachusetts General Hospital, Boston, MA 02114, United States

d Department of Genetics, Harvard Medical School, Boston, MA 02115, United States

e Department of Chemistry and Chemical Biology, Harvard University, Cambridge, MA 02138, United States

f Howard Hughes Medical Institute, Department of Chemistry, University of Chicago, Chicago, IL 60637, United States

**To whom correspondence should be addressed: jwszostak@uchicago.edu*

Table of Contents

Page 2	Materials
Page 3	Experimental Conditions for Figures 2 and 3: ^{31}P NMR Time Course
Page 4	Primer Labeling Experimental Conditions
Page 5	Experimental Conditions for Figure 4D: Positive Control RNA Copying
Page 6	Standard Experimental Conditions for In Situ Activation and RNA Copying
Page 7	Experimental Conditions for Figure 6
Page 8	Experimental Conditions for Figure 7C
Pages 9-10	Supplementary Figures

Materials and Methods

Materials. Reagents and solvents were obtained at the highest purity available from Acros Organics, Alfa Aesar, Fisher Scientific, Sigma-Aldrich, ThermoFisher Scientific, or Tokyo Chemical Industry Co., and were used without any further purification unless noted below. Nucleoside-5′-monophosphate, free acid, was purchased from Santa Cruz Biotechnology. 2-Aminoimidazole hydrochloride and 2,2′-dipyridyldisulfide were purchased from Combi Blocks. RNA oligonucleotides were purchased from Integrated DNA Technologies. All reactions were carried out in DNase/RNase-free distilled water.

Stock solutions of nucleoside-5′-monophosphate disodium salt, 2-aminoimidazole hydrochloride, and 2-[4-(2- hydroxyethyl)piperazin-1-yl]ethanesulfonic acid (HEPES) buffer, were prepared by dissolving the corresponding reagent in DNase and RNase-free distilled water. The exact concentrations of the nucleoside-5′-monophosphates were determined by analysis of serial dilutions on a spectrophotometer. The absolute concentrations of the other stock solutions were found by comparing the integrals of ^{1}H NMR peaks of interest to the calibrant, adenosine-5′-monophosphate by NMR spectroscopy.

^{1}H and ^{31}P NMR spectra were obtained using a 400 MHz NMR spectrometer (Varian INOVA) operating at 400 MHz and 161 MHz respectively. Chemical shifts (δ) are shown in ppm. pH values were determined by a micro pH probe (Orion 9863BN) equipped with a needle tip and a SevenCompact meter (Mettler Toledo S220). All spectra were analyzed using MestReNova (version 12.0.3). The yields of conversion were determined by the relative integration of the signals in the ^{1}H or ^{31}P NMR spectra.

Experimental Conditions for Figures 2 and 3: ^{31}P NMR Time Course

Step 1

A solution of adenosine-5′-monophosphate, 2-aminoimidazole, nucleophilic organocatalyst, $MgCl_2$, HEPES buffer (pH = 8), 2-methylbutyraldehyde, and deuterium oxide was prepared (500 µL total volume), and the mixture vortexed vigorously. The final concentrations were as follows:

**Reagent**	**Concentration**
AMP	20 mM
2AI	10 mM
organocatalyst	50 mM
$MgCl_2$	30 mM
HEPES (pH = 8)	200 mM
D_2O	10% *w*/*w*
aldehyde	200 mM
MeNC	200 mM

Step 2

Methyl isocyanide (14 M stock solution, 7.1 µL, 200 mM final concentration) was added to the above solution, initiating the reaction. The reaction mixture was then vortexed for 30 s before being transferred to an NMR tube. The progress of the reaction was monitored by ^{31}P NMR spectroscopy over the course of 8 h. The entire time-course of the reaction was obtained through arrayed acquisition and tracked by monitoring peak areas.

Primer Labeling Experimental Conditions

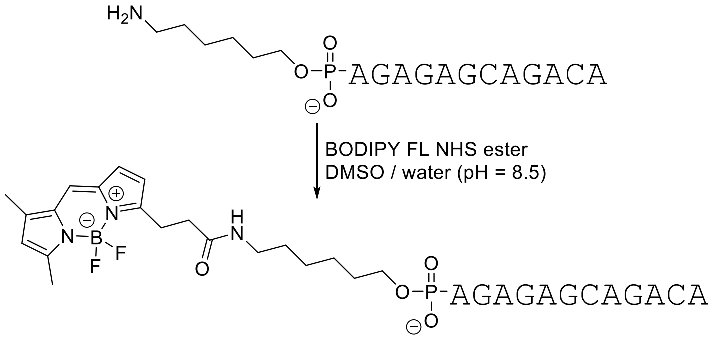

BODIPY FL labeled primer

To RNA oligonucleotide 5′-hexylamino-AGAGAGCAGACA-3′ (1 mM, 5 µL) and sodium borate buffer (pH = 8.5, 1 M, 5 µL) in water (27.5 µL) was added a solution of BODIPY™ FL NHS ester (10 mM in DMSO, 12.5 µL). The mixture was vortexed, sealed in a blackened flask, and incubated for 16 h. The oligonucleotide was then precipitated with NH_4OAc (5 M, 40 µL) and isopropanol (600 µL), and cooled on dry ice for 10 min. The mixture was centrifuged at 15000 rpm for 5 min and the dark orange pellet was washed with 80% ethanol (2 × 400 µL) and dried *in vacuo*. Resuspending the solid product in water (20 µL) afforded a bright green solution of BODIPY FL dye labeled primer (195 µM) which was used without further purification.

Experimental Conditions for Figure 4D: Positive Control RNA Copying

Step 1

A solution of labeled primer 5′-BODIPY FL-AGAGAGCAGACA-3′ (30 μM), template 5′- CCGUGUCUGCUCUCUAAAAA-3′ (45 μM), and HEPES buffer (pH = 8, 20 mM) was annealed by heating at 90 °C for 1 min followed by cooling to 23°C at a rate of 0.1 °C/s.

Step 2

A solution of pre-annealed primer/template duplex, $MgCl_2$, and HEPES buffer (pH = 8), was prepared. The experiment was initiated upon the addition of 2AI-activated phosphorimidazolides *G and *C (20 µL total volume). The final concentrations were as follows:

**Reagent**	**Concentration**
Primer	2 μM
Template	3 μM
*C	10 mM
*G	10 mM
$MgCl_2$	30 mM
HEPES (pH = 8)	200 mM

Step 3

After 24 h an aliquot (1.5 µL) was removed and mixed with quenching buffer (13.5 µL) containing EDTA (pH = 8.0, 30 mM), complementary RNA (20 µM), and 90% (v/v) formamide. The sample was heated at 90 °C for 1 min, and a 5 µL aliquot was separated by 20% (19:1) denaturing PAGE with 7 M urea. The gel was scanned using a Typhoon 9410 scanner, and the bands were quantified using the ImageQuant TL software.

Standard Experimental Conditions for *In Situ* Activation and RNA Copying

Step 1

A solution of labeled primer 5′-BODIPY FL-AGAGAGCAGACA-3′ (30 μM), template 5′- CCGUGUCUGCUCUCUAAAAA-3′ (45 μM), and HEPES buffer (pH = 8, 20 mM) was annealed by heating at 90 °C for 1 min followed by cooling to 23°C at a rate of 0.1 °C/s.

Step 2

A solution of pre-annealed primer/template duplex, cytidine-5′-monophosphate, guanosine-5′-monophosphate, 2-aminoimidazole, nucleophilic organocatalyst, $MgCl_2$, 2-methylbutyraldehyde, and HEPES buffer (pH = 8) was prepared (40 μL total volume). The final concentrations were as follows:

**Reagent**	**Concentration**
Primer	2 μM
Template	3 μM
CMP	10 mM
GMP	10 mM
2AI	10 mM
organocatalyst	50 mM
$MgCl_2$	30 mM
HEPES (pH = 8)	200 mM
aldehyde	200 mM
MeNC	200 mM

Step 3

Methyl isocyanide (14 M stock solution, 0.57 μL, 200 mM final concentration) was added to the above solution, initiating the reaction. The reaction mixture was then vortexed for 30 s and incubated in the dark for 24 h. An aliquot (1.5 μL) was removed and mixed with quenching buffer (13.5 μL) containing EDTA (pH = 8.0, 30 mM), complementary RNA (20 μM), and 90% (v/v) formamide. The sample was heated at 90 °C for 1 min, and a 5 μL aliquot was separated by 20% (19:1) denaturing PAGE with 7 M urea. The gel was scanned using a Typhoon 9410 scanner, and the bands were quantified using the ImageQuant TL software.

Experimental Conditions for Figure 6: ^{31}P NMR Time Course of Periodic Reactivation of AMP

Step 1

A solution of adenosine-5′-monophosphate, 2-aminoimidazole, 2-aminothiazole, 4,5-dicyanoimidazole, 4-aminopyrimidine, $MgCl_2$, HEPES buffer (pH = 8), and deuterium oxide was prepared (2200 µL total volume) and the mixture was vortexed vigorously then cooled to −3 °C. The final concentrations were as follows:

**Reagent**	**Concentration**
AMP	40 mM
2AI	30 mM
2AT	20 mM
DCI	20 mM
4-aminopyrimidine	20 mM
$MgCl_2$	30 mM
HEPES (pH = 8)	200 mM
D_2O	10% *w*/*w*
aldehyde	200 mM
MeNC	200 mM

Step 2

2-Methylbutyraldehyde (47 µL, 200 mmol final concentration) was added to the above solution, and the mixture was vortexed vigorously. Methyl isocyanide (12.5 M stock solution, 35 µL, 200 mM final concentration) was added to the above mixture, initiating the reaction. The reaction mixture was then vortexed for 30 s before an aliquot (500 µL) was transferred to a cooled NMR tube, while the remaining mixture was sealed in a blackened vial and incubated at −3 °C. The progress of the reaction was monitored by ^{31}P NMR spectroscopy over the course of 24 h, using a probe cooled to −3 °C. The entire time-course of the reaction was obtained through arrayed acquisition and tracked by monitoring peak areas.

Step 3

Step 2 was repeated three times, with a further 200 mM portion of 2-methylbutyraldehyde and methyl isocyanide added to the reaction mixture every 24 h, followed by the transfer of aliquots (500 µL each) for ^{31}P NMR analysis. These further additions required the following volumes of reagents:

**Day**	**2MBA**	**MeNC**
1	36 µL	27 µL
2	25 µL	18.4 µL
3	12.8 µL	9.6 µL

Experimental Conditions for Figure 7C: *In Situ* Activation and RNA Copying

Step 1

A solution of labeled primer 5′-BODIPY FL-AGAGAGCAGACA-3′ (30 µM), template 5′- GCCUCAUUCUGCUCUCU-3′ (45 µM), and HEPES buffer (pH = 8, 20 mM) was annealed by heating at 90 °C for 1 min followed by cooling to 23°C at a rate of 0.1 °C/s.

Step 2

A solution of pre-annealed primer/template duplex, helper trimers, nucleoside-5′-monophosphates, 2-aminoimidazole, 2-aminothiazole, 4,5-dicyanoimidazole, 4-aminopyrimidine, $MgCl_2$, and HEPES buffer (pH = 8), was prepared (50 µL total volume) and the mixture was vortexed vigorously then cooled to −3 °C. The final concentrations were as follows:

**Reagent**	**Concentration**
Primer	2 µM
Template	3 µM
AMP	10 mM
CMP	10 mM
GMP	10 mM
UMP	10 mM
pUGA	500 µM
pGAG	500 µM
pAGG	500 µM
pGGC	500 µM
pGCG	500 µM
pCGC	500 µM
2AI	30 mM
2AT	20 mM
DCI	20 mM
4-aminopyrimidine	20 mM
$MgCl_2$	30 mM
HEPES (pH = 8)	200 mM
aldehyde	200 mM
MeNC	200 mM

Step 3

2-Methylbutyraldehyde (1.1 µL, 200 mmol final concentration) was added to the above solution, and the mixture was vortexed vigorously. Methyl isocyanide (14 M stock solution, 0.70 µL, 200 mmol final concentration) was added, initiating the reaction. The reaction mixture was then vortexed for 30 s and incubated in the dark for 24 h at −3 °C. An aliquot (1.7 µL each) was removed and mixed with quenching buffer (13.5 µL) containing EDTA (pH = 8.0, 30 mM), complementary RNA (20 µM), and 90% (v/v) formamide. The sample was heated at 90 °C for 1 min, and a 5 µL aliquot was separated by 20% (19:1) denaturing PAGE with 7 M urea. The gel was scanned using a Typhoon 9410 scanner, and the bands were quantified using the ImageQuant TL software.

Step 4

Step 3 was repeated 5 times, with a further 200 mM portion of 2-methylbutyraldehyde (1.1 µL each) and methyl isocyanide (0.70 µL each) added to the reaction mixture every 24 h, followed by the removal of an aliquot (1.7 µL) for PAGE analysis.

Supplementary Figures

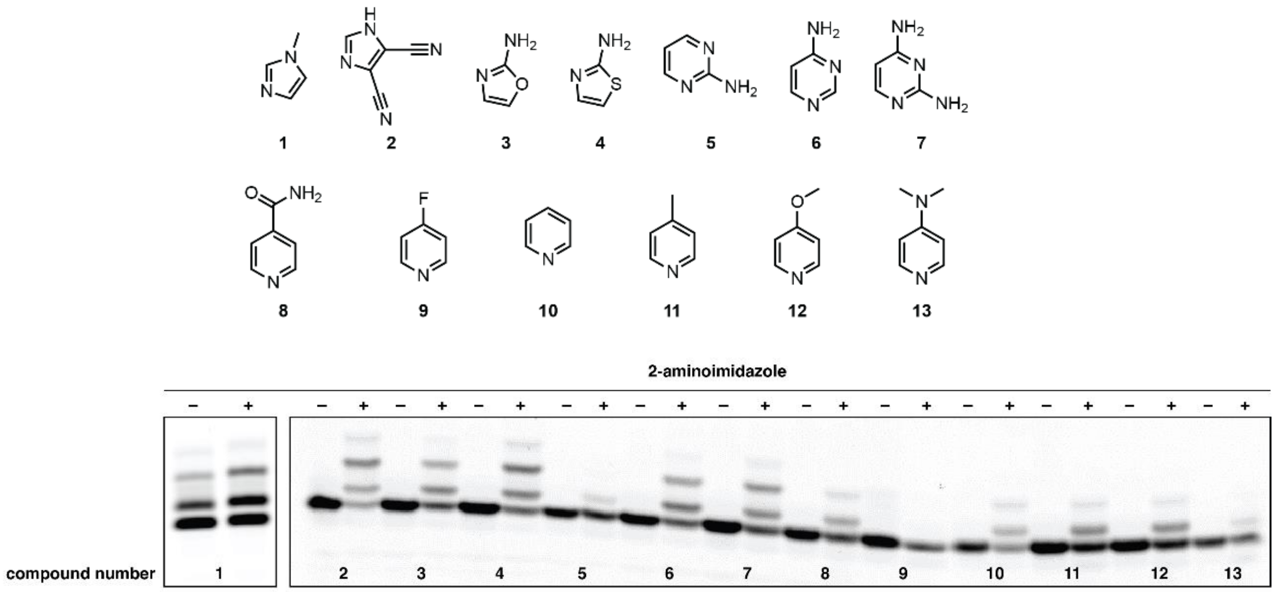

Figure S1. Gel data for *in situ* activation and RNA copying catalyzed by heteroaromatic small molecules, corresponding to table 1. Standard conditions: CMP (10 mM), GMP (10 mM), 2AI (10 mM), organocatalyst (50 mM), $MgCl_2$ (30 mM), HEPES (200 mM, pH = 8), initiated with MBA/MeNC (200 mM each); time = 24 h.

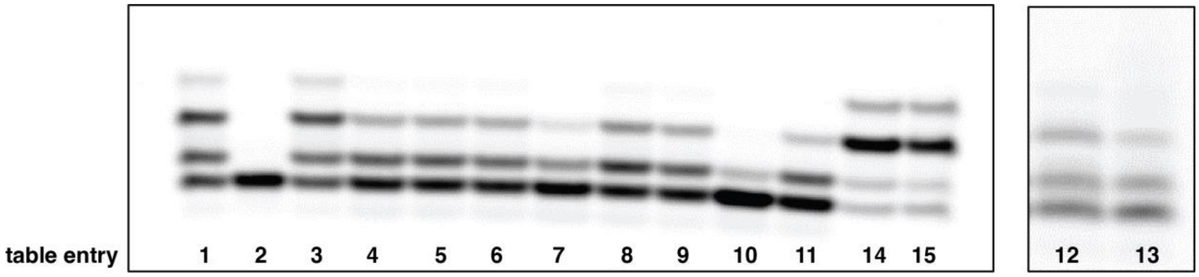

Figure S2. Gel data for conditions screen for *in situ* activation and copying catalyzed by 2AT/DCI/4-aminopyrimidine (1:1:1), corresponding to table 2. Standard conditions: CMP (10 mM), GMP (10 mM), 2AI (10 mM), 2AT/DCI/4-aminopyrimidine (1:1:1, 50 mM total), $MgCl_2$ (30 mM), HEPES (200 mM, pH = 8), initiated with MBA/MeNC (200 mM each). time = 24 h.

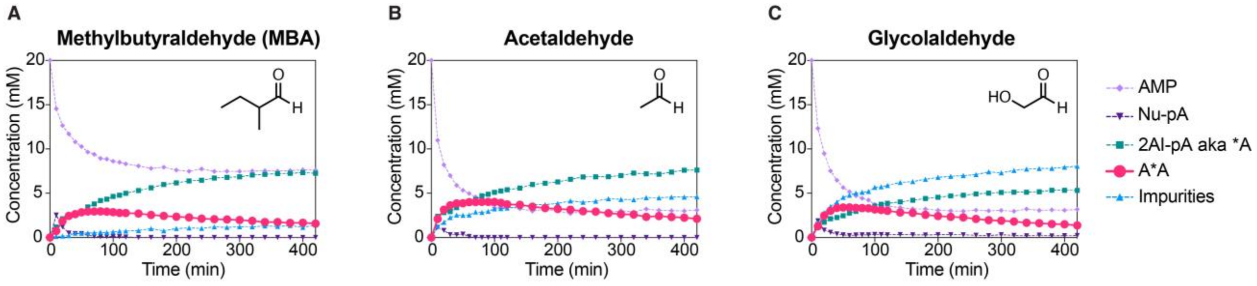

Figure S3. ^{31}P NMR time course charts for Passerini-type adenosine monophosphate activation catalyzed by 2AT/DCI/4-aminopyrimidine with various aldehydes. *Conditions:* AMP (20 mM), 2-AI (10 mM), 2AT/DCI/4-aminopyrimidine (1:1:1, 50 mM total), $MgCl_2$ (30 mM), HEPES (200 mM, pH = 8), initiated with aldehyde/MeNC (200 mM each). A: MBA; B: acetaldehyde; C: glycolaldehyde.

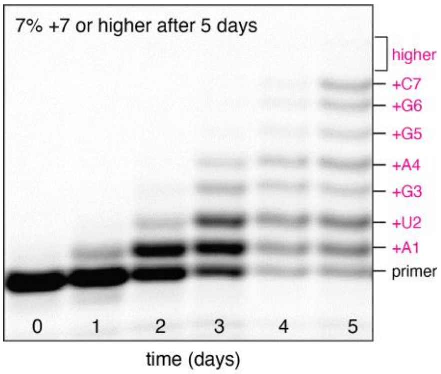

Figure S4. Helper trimer assisted RNA copying with a template containing all four canonical nucleotides at room temperature, driven by periodic reactivation of NMPs and trimers. *Conditions:* NMPs (10 mM each), 2AI (30 mM), catalyzed by 2AT/DCI/4-aminopyrimidine (1:1:1, 60 mM total) with MBA/MeNC (200 mM each, every 24 h) at room temperature.

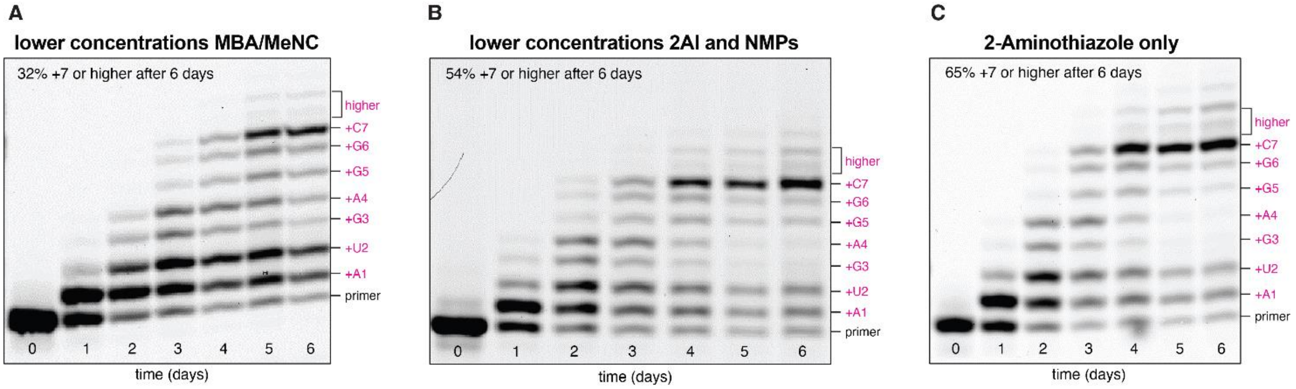

Figure S5. Helper trimer assisted RNA copying with a template containing all four canonical nucleotides, driven by periodic reactivation of NMPs and trimers at −3 °C. A: NMPs (10 mM each), 2AI (30 mM), catalyzed by 2AT/DCI/4-aminopyrimidine (1:1:1, 60 mM total) with MBA/MeNC (100 mM each, every 24 h); B: NMPs (5 mM each), 2AI (15 mM), catalyzed by 2AT/DCI/4-aminopyrimidine (1:1:1, 60 mM total) with MBA/MeNC (200 mM each, every 24 h); C: NMPs (10 mM each), 2AI (30 mM), catalyzed by 2AT (60 mM) with MBA/MeNC (200 mM each, every 24 h).
